# The incidence and risk factors of intraoperative bacterial contamination in primary total knee arthroplasty

**DOI:** 10.3389/fsurg.2024.1458403

**Published:** 2024-11-08

**Authors:** Lijun Xing, Fengyue Liu, Enrun Li, Yuling Kang, Kunyuan Tan, Juhong Li

**Affiliations:** ^1^ Operating Theatre, Peking University International Hospital, Beijing, China; ^2^ Department of Anesthesiology, The Fourth Medical Center of the PLA General Hospital, Beijing, China

**Keywords:** incidence, risk factor, intraoperative bacterial contamination, total knee arthroplasty, fever, leukocytosis

## Abstract

**Background:**

Infection is a devasting complication after arthroplasty. Identifying potential sources contributing to intraoperative bacterial transmission can help to reduce surgical-site infections.

**Objective:**

The aim of this study was to identify the incidence and risk factors of intraoperative bacterial contamination (IBC) in primary total knee arthroplasty (TKA) procedures.

**Methods:**

Active surveillance cultures were used to detect IBC from 125 consecutive unilateral primary TKAs. The cultures were taken from different sites (surgical instruments, gloves of surgeons and surgical incisions) at different time points (before surgery, 30 min and at the end of the surgery) during arthroplasty procedures. Patient characteristics, including age, height, body weight, body mass index, comorbidity of diabetes mellitus, operative duration, intraoperative blood loss, types of prophylactic antibiotics were recorded. The white blood cell level before, the 3rd and 7th day after surgery were measured and recorded. In addition, patients were also followed for fever and surgical-site infections within 14 days after surgery.

**Results:**

In total, 1,000 cultures were taken. 91 (9.1%) of them showed an IBC. None of bacterial cultures of gloves and instruments before surgery were positive. At 30 min from the beginning of the procedure, 29 cultures (7.7%) from 16 patients (12.8%) showed a contamination. At the end of the surgery, 62 cultures (16.5%) from 32 patients (25.6%) showed a contamination. There were 15 cases (12.0%) of fever within 14 days after surgery, of which 11 cases with IBC, and 4 cases without IBC. No postoperative surgical-site infection occurred in all consecutive unilateral primary TKAs. The binomial logistic regression analyses confirmed that operative duration was the risk factor of IBC [OR 1.137 (95% CI 1.023 to 1.322), *p* = 0.014]. Moreover, compared to control group, the patients with IBC had a greater change of white blood cell level in the 3rd day after surgery (*p* = 0.022), and a higher risk of fever within 14 days after surgery (*p* < 0.001).

**Conclusion:**

The bacterial contamination rate during primary TKA is relatively high, despite the practice of standard preventive measures. Intraoperative bacterial contamination increases with long operating time, which may be one of the factors contributing to fever and leukocytosis after surgery.

## 1 Introduction

Periprosthetic joint infection (PJI) is a devasting and extremely challenging complication following arthroplasty, which results in heavy costs to the healthcare system (1). Despite several preventive procedures, including hand hygiene, preoperative patient skin antisepsis, prophylactic administration of antibiotics, and intrawound lavage with a large amount of saline, a risk of infection after arthroplasty remains (2). The reported 90-day PJI rates are approximately 1.5–2.2% and 1.3%–1.7% following total knee arthroplasty (TKA) and total hip arthroplasty, respectively (3).

The pathogenic bacteria causing the infection may be endogenous or exogenous. Wound bacterial contamination during or at the end of arthroplasty is assumed to be a cause of early postoperative infections, also known as surgical site infection (SSI) (4). The pathogenic bacteria frequently associated with SSIs include Staphylococcus aureus, coagulase-negative staphylococci, Enterococcus spp, and Escherichia coli, which can be transferred via direct or indirect contact (5).

It is the surgeon's responsibility to identify and reduce the risk of SSI, and to be aware of potential sources of intraoperative bacterial contamination (IBC) during arthroplasty. Surgical instruments, gloves of surgeons, and surrounding areas of the surgical field can all be potential sources of IBC during arthroplasty (6). It is to mention, to the authors’ knowledge, that this is the first study investigating the incidence and risk factors of IBC simultaneously at different sites (surgical instruments, gloves of surgeons and surgical incisions) at different time points (before surgery, 30 min and at the end of the surgery) during arthroplasty procedures. Therefore, detecting the incidence of bacterial contamination in these different sites at different time points during arthroplasty procedures, and analyzing the risk factors of IBC, are the preconditions for achieving better prevention of SSIs and PJI. The aim of this study was to identify the incidence and risk factors of IBC in primary TKA procedures.

## 2 Materials and methods

### 2.1 Study population

This prospective observational study was approved by the institutional review board of the authors’ affiliated institutions (2020-KY-0025). Written informed consent was obtained from all participants. From February 2021 to September 2022, 125 consecutive patients diagnosed with severe knee osteoarthritis who were treated with primary unilateral TKA, were identified for this observational study. Patients with previous trauma around the surgical site, those with a history of infection in the knee, and revision surgery cases were excluded from the study.

### 2.2 Treatment algorithm

All aseptic arthroplasty procedures were carried out in a standardised manner with 1.5 g of prophylactic cefuroxime sodium or 0.6 g of prophylactic clindamycin phosphate administered prior to the skin incision in an operation theatre with laminar flow. Prophylactic antibiotics were administered intravenously for 24 h after surgery. The procedures were performed by one senior surgeon. All patients (125 knees) were implanted with the A3 fix-bearing, posterior-stabilized total knee system (AK Medical, Beijing, China). All procedures were also performed using the same surgical instruments. During arthroplasty, the surgical staff (surgeon, assisting-surgeon) wore water-resistant sterile surgical gowns, 2 pairs of gloves. Tourniquet was used for the entire procedure. Preoperative skin preparation was performed with 3% iodine tincture once, and then 75% alcohol was used for deiodination twice. A skin drape with the povidone-iodine was applied on the skin before the incision. The suction drain placed intraoperatively was removed, and patients were allowed to ambulate within 24 h after surgery.

### 2.3 Data collection

The cultures were collected with a swab from different sites (surgical instruments, gloves of surgeons and surgical incisions) at different time points (before surgery, 30 min and at the end of the surgery) during arthroplasty procedures. The surgical instrument sampled was the front end of the oscillating saw after performing the osteotomy, and a swab was used to directly wipe off the bone debris on the surface of the oscillating saw. Sample from the surgeon's glove was collected from the palmar distal end of the surgeon's right index finger, and the surface was wiped with a swab. Culture sample of surgical incisions was collected from the subcutaneous tissue at the edge of the surgical incisions with a sterile swab. Therefore, culture samples were not obtained from surgical incisions before surgery. In the microbiology lab, these samples were rolled onto sheep blood agar and MacConkey plates for a minimum of 20 s. Robertson cooked meat medium was used afterwards for the cultivation of organisms. The aforementioned plates and medium were incubated in 5% carbon dioxide for 72 h and 7 days to allow for aerobic and anaerobic bacterial growth, respectively. Bacterial contamination was judged by the results of microscopic examination with gram stain and colony morphology. IBC was defined as a positive culture at any site from different sites (surgical instruments, gloves of surgeons and surgical incisions). The time points selected for this study were before surgery, 30 min and at the end of the surgery.

Patient characteristics, including age, height, body weight, body mass index, comorbidity of diabetes mellitus, operative duration, intraoperative blood loss, types of prophylactic antibiotics were recorded. The white blood cell level before, the 3rd and 7th day after surgery were measured and recorded. In addition, patients were also followed for fever and surgical-site infections within 14 days after surgery.

### 2.4 Statistical analysis

All data were analyzed using GraphPad Prism (Version 9; GraphPad Software, USA). Continuous data were presented as a mean ± standard deviation. The Shapiro–Wilk test was used to test continuous variables for normal distribution. For continuous variables with a normal distribution, the comparison between groups was conducted using independent samples *t*-test or paired samples *t*-test; for continuous variables with non-normal distribution, the comparison between groups was performed using the Mann-Whitney *U* test. Categorical variables were compared between groups using Fisher's exact test. The values were considered significant when the two-tailed *p* value of the difference was less than 0.05.

## 3 Results

### 3.1 Patient characteristics

125 consecutive unilateral primary TKAs were enrolled in this study, which included 10 men and 115 women with a median age of 67 (64.0–71.0) years. Demographic characteristics of the patients are shown in Table 1. Overall, we identified 62 cultures (16.5%) from 32 patients (25.6%) showed a contamination during arthroplasty procedures.

**Table 1:** Patient characteristics.

	Total study cohort (*n* = 125)
Age (years), median (IQR)	67.0 (64.0–71.0)
Females	115 (92%)
BMI (kg/m^2^), mean (SD)	27.3 (3.2)
WBC (10^9^/L) before surgery, median (IQR)	6.4 (5.2–7.3)
WBC (10^9^/L) at day 3 after surgery, median (IQR)	9.2 (7.9–11.4)
WBC (10^9^/L) at day 7 after surgery, median (IQR)	7.0 (6.0–9.3)
Prophylactic antibiotics (Cefuroxime sodium/Clindamycin phosphate)	122/3
Duration of operation (mins), mean (SD)	88.4 (15.1)
Intraoperative blood loss (ml), median (IQR)	50 (20–100)
Diabetes mellitus	44 (35.2%)
Fever (with IBC/without IBC)	15 (11/4)
Surgical-site infection	None
IBC (30 min), cultures/patients	29 (7.7%)/16 (12.8%)
IBC (end of surgery), cultures/patients	62 (16.5%)/32 (25.6%)

IQR, inter-quartile rang; BMI, body mass index; SD, standard deviation; WBC, white blood cell; IBC, intraoperative bacterial contamination. Values are given as absolute numbers (percentage), if not otherwise specified.

### 3.2 The incidence of intraoperative bacterial contamination

In total, 1,000 cultures (375 from gloves of surgeons; 375 from surgical instruments; 250 from surgical incisions) were taken. 91 (9.1%) of them showed an IBC. None of bacterial cultures of gloves and instruments before surgery were positive. At 30 min of the surgery, 29 cultures (7.7%) from 16 patients (12.8%) showed a contamination, of which 10 (8.3%) of 125 cultures taken from the surgical instruments showed a contamination; 13 (10.4%) of 125 cultures taken from the gloves of surgeons showed a contamination; while 6 (4.8%) of 125 cultures taken from the surgical incisions showed a contamination. At the end of the surgery, 62 cultures (16.5%) from 32 patients (25.6%) showed a contamination, of which 22 (17.6%) of 125 cultures taken from the surgical instruments showed a contamination; 27 (21.6%) of 125 cultures taken from the gloves of surgeons showed a contamination; while 13 (10.4%) of 125 cultures taken from the surgical incisions showed a contamination. The incidence of IBC from different sites (surgical instruments, gloves of surgeons and surgical incisions) at different time points (before surgery, 30 min and at the end of the surgery) during arthroplasty procedures is detailed in Table 2.

**Table 2:** The incidence of IBC from different sites at different time points.

	Surgical instruments (*n* = 375)	Gloves of surgeons (*n* = 375)	Surgical incisions (*n* = 250)
Before surgery (*n* = 250)	0 (0.0%)	0 (0.0%)	NA
30 min of the surgery (*n* = 375)	10 (8.3%)	13 (10.4%)	6 (4.8%)
End of the surgery (*n* = 375)	22 (17.6%)	27 (21.6%)	13 (10.4%)

IBC, intraoperative bacterial contamination; NA, not applicable. Values are given as absolute numbers (percentage).

We also recorded the distribution of isolated organisms in 91 IBC during arthroplasty procedure. Table 3 shows the distribution of the different bacteria. The microbiology profile in this study also matched those reported in the literature with staphylococcus aureus and staphylococcus epidermidis as the most common contaminating bacteria in TKA.

**Table 3:** Distribution of isolated organisms in IBC.

Bacteria	*n* *=* 91
Staphylococci epidermidis	41 (45.1%)
Staphylococci saprophyticus	35 (38.5%)
Escherichia coli	10 (11.0%)
Staphylococcus haemolyticus	2 (2.2%)
Micrococcus luteus	1 (1.1%)
Kocuria kristinae	1 (1.1%)
Staphylococcus saprophyticus	1 (1.1%)

IBC, Intraoperative bacterial contamination. Values are given as absolute numbers (percentage).

### 3.3 Effect of IBC on postoperative fever and leukocytosis after joint arthroplasty

**Figure 1:**
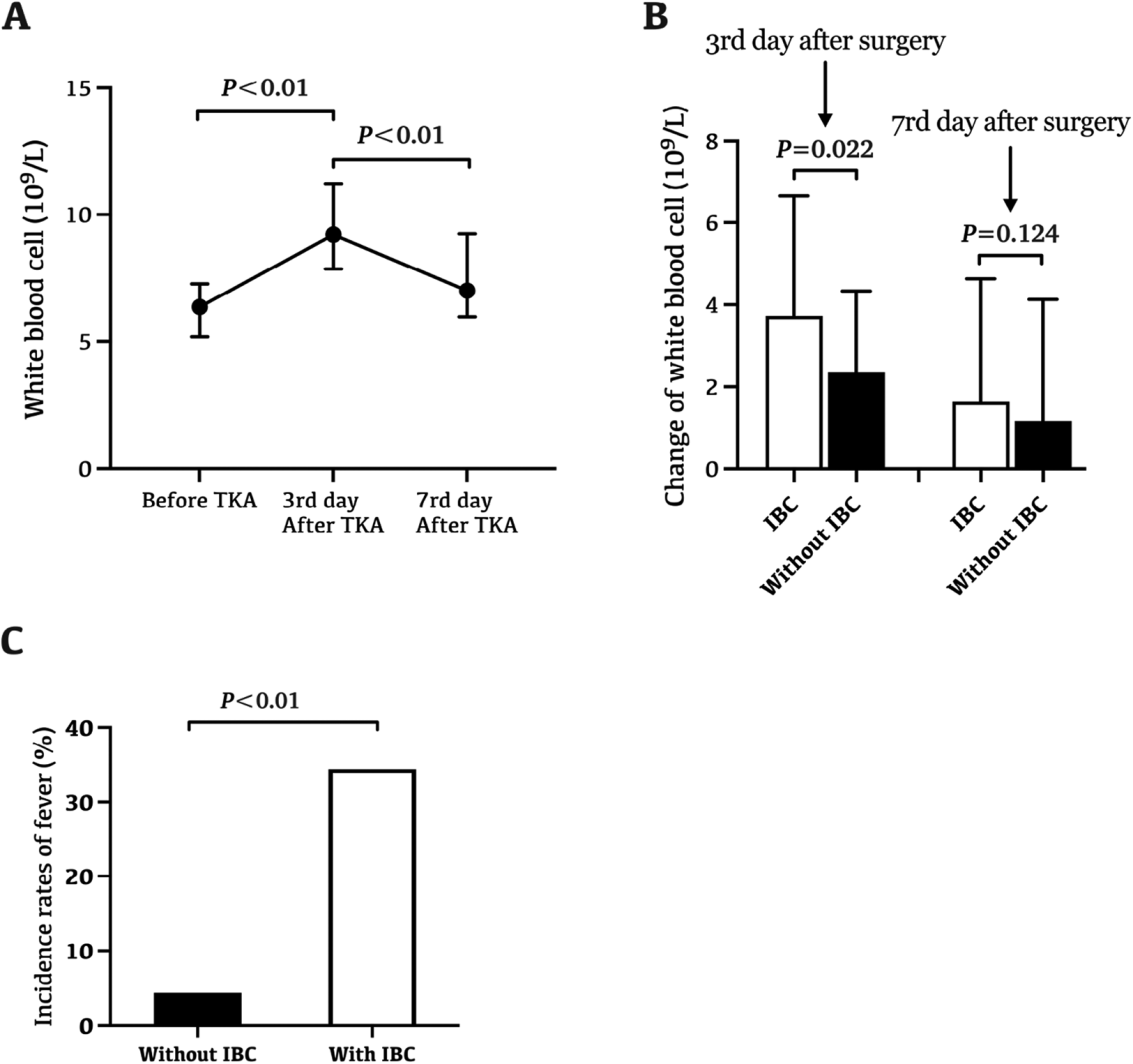
The influence of IBC on changes of WBC level and postoperative fever. **(A)** The change trend of WBC level in perioperative period. The white blood cell (WBC) level before surgery was 6.4 (5.2–7.3) 10^9^/L, which increased to 9.2 (7.9–11.4) 10^9^/L at day 3 after surgery, and decreased to 7.0 (6.0–9.3) 10^9^/L at day 7 after surgery. **(B)** Patients with IBC during arthroplasty procedures showed a more significant increase in WBC at day 3 after surgery (*P* = 0.022). **(C)** Patients with IBC during arthroplasty procedures showed a higher risk of fever within 14 days after surgery (*P* < 0.01). There were 15 cases (12.0%) of fever within 14 days after surgery, of which 11 cases with IBC, and 4 cases without IBC.

The white blood cell (WBC) level before surgery was 6.4 (5.2–7.3) 10^9^/L, which increased to 9.2 (7.9–11.4) 10^9^/L at day 3 after surgery, and decreased to 7.0 (6.0–9.3) 10^9^/L at day 7 after surgery (Figure 1A). Patients with IBC during arthroplasty procedures showed a more significant increase in WBC at day 3 after surgery (Figure 1B, *P* = 0.022). Similarly, there were 15 cases (12.0%) of fever within 14 days after surgery, of which 11 cases with IBC, and 4 cases without IBC. Patients with IBC during arthroplasty procedures showed a higher risk of fever within 14 days after surgery (Figure 1C, *P* < 0.01). However, no postoperative surgical-site infection occurred in all consecutive unilateral primary TKAs.

### 3.4 The risk factors of IBC in primary TKA

The binomial logistic regression analyses confirmed that operative duration was the risk factor of IBC [OR 1.137 (95% CI 1.023 to 1.322), *p* = 0.014]. However, age, sex, height, body weight, body mass index, comorbidity of diabetes mellitus, intraoperative blood loss, and types of prophylactic antibiotics were not risk factors of IBC during arthroplasty procedures with the binomial logistic regression analyses (Table 4).

**Table 4:** Binomial logistic regression analysis of risk factors for intraoperative bacterial contamination during arthroplasty.

Parameter	IBC (*n* = 39)	No IBC (*n* = 86)	OR (95% CI)	*p* value
Age (years), median (IQR)	68.0 (64.0–73.0)	66.0 (65.0–72.0)	1.101 (0.330–3.197)	0.865
Females	35 (89.7%)	80 (93.0%)	0.896 (0.382–2.102)	0.797
BMI (kg/m^2^), mean (SD)	28.1 (2.9)	27.2 (3.7)	1.014 (0.989–1.041)	0.285
Prophylactic antibiotics with cefuroxime	38 (97.4%)	84 (97.7%)	1.004 (0.996–1.006)	0.927
Duration of operation (mins), mean (SD)	95.2 (18.1)	84.0 (11.6)	1.137 (1.023–1.322)	0.014*
Intraoperative blood loss (ml), median (IQR)	50 (20–100)	50 (20–100)	1.003 (0.230–3.995)	0.953
Diabetes mellitus	16 (41.0%)	28 (32.6%)	1.451 (0.505–3.865)	0.199

IBC, Intraoperative bacterial contamination. Values are given as absolute numbers (percentage), if not otherwise specified.

* Statistical significance, *p* < 0.05.

## Discussion

PJI after arthroplasty is a devastating complication, which is the most common cause behind revision procedures (1, 7, 8). IBC during arthroplasty procedures is assumed to be a cause of early postoperative infections, which may induce the occurrence of postoperative PJI, or increase the risk of rerevision for PJI after aseptic revision TKA (9, 10). The most important finding of this study is that IBC occurred in cases with a longer operative duration, which may be one of the risk factors contributing to fever and leukocytosis after surgery. It is to mention, to the authors’ knowledge, that this is the first study investigating the incidence and risk factors of IBC in different sites (surgical instruments, gloves of surgeons and surgical incisions) at different time points (before surgery, 30 min and at the end of the surgery) during arthroplasty procedures.

Despite practicing standard precautions to minimize IBC in hip and knee arthroplasty, it has been reported a high incidence for surgical field contaminated, and the incidence of IBC varies among different studies (11–13). So, it is very important for surgeons to be aware of all potential contaminated areas in the operating room. Several IBC rates and potential contaminated areas have been reported. For instance, around 15.2% of the surgeons’ glove fingertips in joint replacement procedures are contaminated (14). It is estimated that the contamination rates in skin and inside blades are approximately 9.4% and 3.2%, respectively, and the intraoperative contamination rates are even higher in suction tips (11.4%), light handles (14.5%) and gloves (28.7%) during surgery (15). Bacterial contamination was identified on 12% of surgical gowns (22% of surgical procedures) during total hip arthroplasty (13). In the current study, the IBC rate in TKA were assessed with 1,000 cultures from different sites at different time points during arthroplasty procedures using microbiological method to ensure accuracy. The final incidence of IBC during TKAs in this study was 62 cultures (16.5%) from 32 patients (25.6%), and IBC mainly occurred in surgical instruments and gloves of surgeons. Meanwhile, the microbiology profile in this study also matched those reported in the literature with staphylococcus aureus and staphylococcus epidermidis as the most common contaminating bacteria in total joint arthroplasty (4, 16, 17).

Identifying risk factors for IBC can aid in risk-stratifying post-operative surveillance. In this study, we collected a variety of patient intrinsic and external factors that may increase IBC, and explored the risk factors for IBC during arthroplasty by the binomial logistic regression analyses. The binomial logistic regression analyses confirmed that operative duration was the risk factor of IBC [OR 1.137 (95% CI 1.023 to 1.322), *p* = 0.014]. Previous studies have also confirmed that a longer operative duration not only increases the amount of blood loss, but also generally increases the risk of IBC in arthroplasty (18). Knee arthroplasty surgeons with shorter median operative durations had a lower risk of SSI than surgeons with typical median operative durations (19). A longer operative duration was an independent predisposing factor for both PJIs and SSIs even after accounting for patient and procedure-related factors (20). Therefore, in joint arthroplasty, we should strive to shorten the operation time, avoid unpacking surgical instruments too early, and shorten the time interval between draping and the surgical incision, so as to reduce the risk of IBC as much as possible.

Fever and leukocytosis are common after joint arthroplasty. After joint arthroplasty, more than half of patients developed leukocytosis, and nearly 15% developed fever (21). IBC may be one cause contributing to fever and leukocytosis in the early stage after arthroplasty. By improving behavioral and environmental parameters, the level of microbial air contamination in the operating room, and the incidence of IBC can be significantly reduced, so as to reduce the occurrence and duration of fever (22). Consistent with previous studies, there were 15 cases (12.0%) of fever within 14 days after surgery, of which 11 cases (34.4%) with IBC, and 4 cases (4.3%) without IBC (18). Those patients with IBC during arthroplasty procedures showed a higher risk of fever within 14 days after surgery, which suggests that IBC may be the main cause of fever after TKAs. Similarly, in this study, patients with IBC during arthroplasty procedures showed a more significant increase in WBC at day 3 after surgery, which suggests that IBC may also be one cause of leukocytosis in the early stage after TKAs.

Our study had a number of limitations. First, this study was performed at a relatively early stage after surgery, although none of our patients, even those with IBC, had an early postoperative infection, the development of PJI is out of this study's scope because of the short duration of follow-up. Another limitation is that we did not investigate the effect of irrigation and lavage, usually performed at end of arthroplasty, on the rate of IBC. Meanwhile, this study was also not meant to address the rate or risk of post-operative infection because of the insufficient sample size.

In conclusion, the bacterial contamination rate during primary TKA is relatively high despite practicing the standard preventive measures. Surgical instruments, gloves of surgeons and surgical incisions can be potential contaminated areas. IBC increases with long operating time, which may be one of the risk factors contributing to fever and leukocytosis after surgery. IBC should not be underestimated during arthroplasty procedures due to the presence of implants. It is very important for surgeons to be aware of all potential contaminated areas in the operating room.

## Data Availability

The raw data supporting the conclusions of this article will be made available by the authors, without undue reservation.
